# Diagnosis, Prognosis, and Management of Breast Cancer in an 81-Year-Old Male Patient

**DOI:** 10.7759/cureus.8277

**Published:** 2020-05-25

**Authors:** Jimmy George, Audrey Albach, Angelica S Robinson, Linden Dixon, Quan D Nguyen

**Affiliations:** 1 Radiology, University of Texas Medical Branch, Galveston, USA

**Keywords:** radiology, breast imaging, breast cancer, elderly, cancer, male breast cancer

## Abstract

Due to the lower rate of breast cancer in men compared to women, there are fewer studies on which to base the treatment of a male patient with breast cancer; and this is further complicated when the patient is part of the elderly population. We report the case of an 81-year-old male who came in for imaging of pulmonary nodules and had an incidental finding of abnormal growth in the breast. Further imaging was performed, and biopsy was completed, confirming invasive ductal carcinoma. Eventually, the patient was treated with a modified radical mastectomy. In this report, we also engage in a discussion of the treatment considerations for patients of male sex and older age group.

## Introduction

Male breast cancer is rare compared to its female counterpart and, naturally, there are fewer studies and data to draw on for its treatment considerations. According to the American Cancer Society, 271,270 new cases of breast cancer were projected to occur in the US in 2019, with male breast cancer accounting for approximately 1% of these diagnoses [1]. However, according to one study that looked at survival after diagnosis of breast cancer from regions across the world, the prognosis for male breast cancer, while similar to that of female breast cancer, was slightly poorer, with male five-year survival being 82.8% and female survival being 88.5% [2]. The same study also noted that the outcome is worse in both sexes for those of older age, especially those over 75 years of age, and for those with the involvement of metastasis.

Acknowledging that prognosis is worse for the elderly as well as that there is a scarcity of data to draw on to make treatment considerations for men, greater care and consideration must be given when examining the literature on how to treat elderly male breast cancer patients. Beyond primary treatment, which is often surgical, proper care must be taken when selecting adjuvant therapy, which is where the data from the literature is really insufficient and lacking in terms of evidence-based treatment options for elderly men. The purpose of this case report is to detail the treatment course of an elderly male patient with breast cancer and discuss literature-based recommendations for adjuvant treatment.

## Case presentation

**Figure 1:**
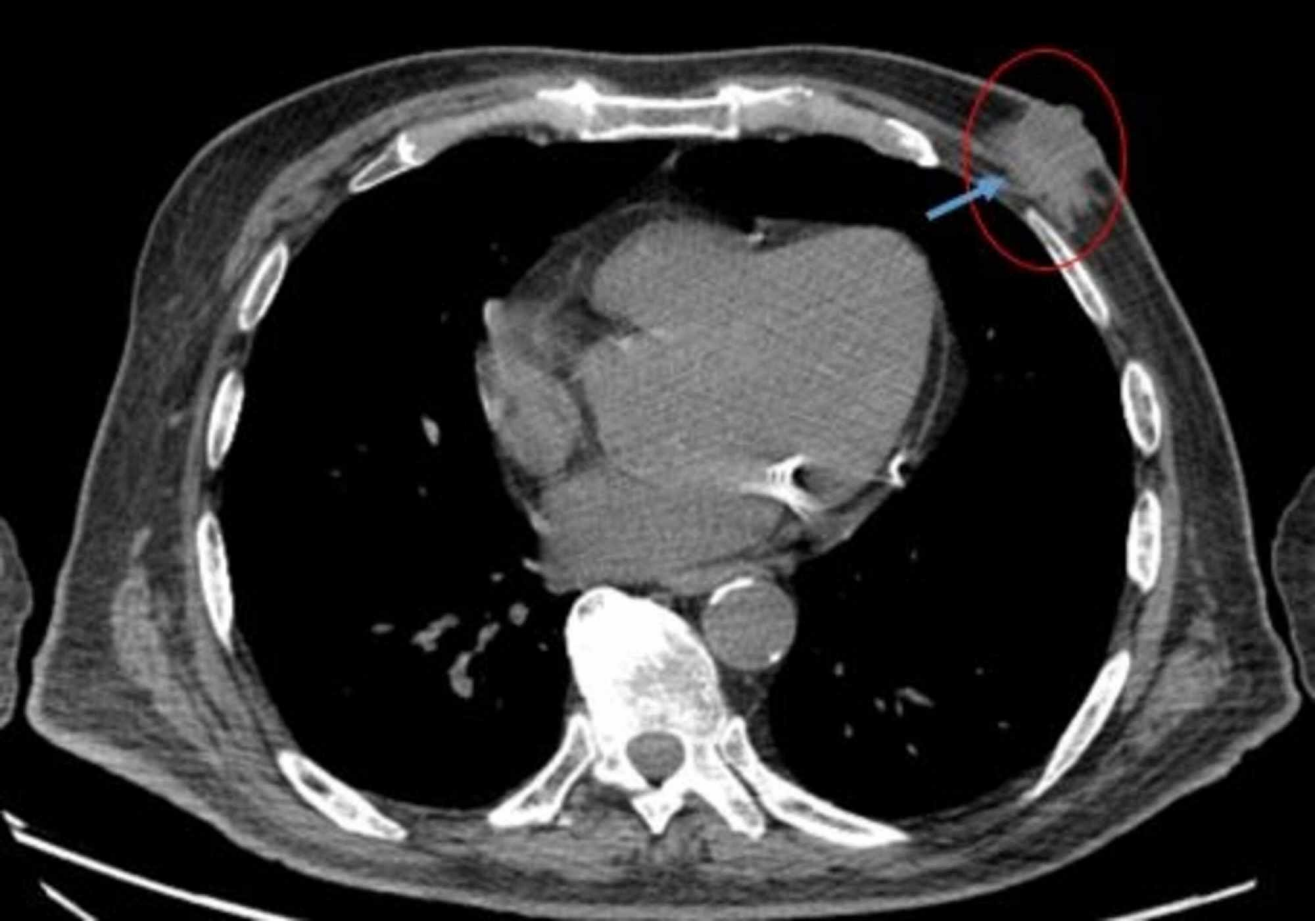
Chest CT of the patient There is a soft tissue density confluent with the left nipple measuring 3.2 cm identified on the image by the blue arrow and red circle. Dedicated breast imaging with mammography and ultrasound is recommended. Multiple subtle lytic lesions are seen in several ribs and left scapula, grossly unchanged and concerning for metastatic disease CT: computed tomography

We present the case of an 81-year-old male with a past medical history of insomnia, peripheral neuropathy, muscle weakness, vascular dementia, vascular parkinsonism, osteoarthritis, and depression. The patient initially presented for CT evaluation of multiple pulmonary nodules, which were ultimately determined to be benign. However, imaging incidentally revealed an abnormal hyperattenuating mass near the left areola (Figure 1), as well as left axillary lymphadenopathy.

**Figure 2:**
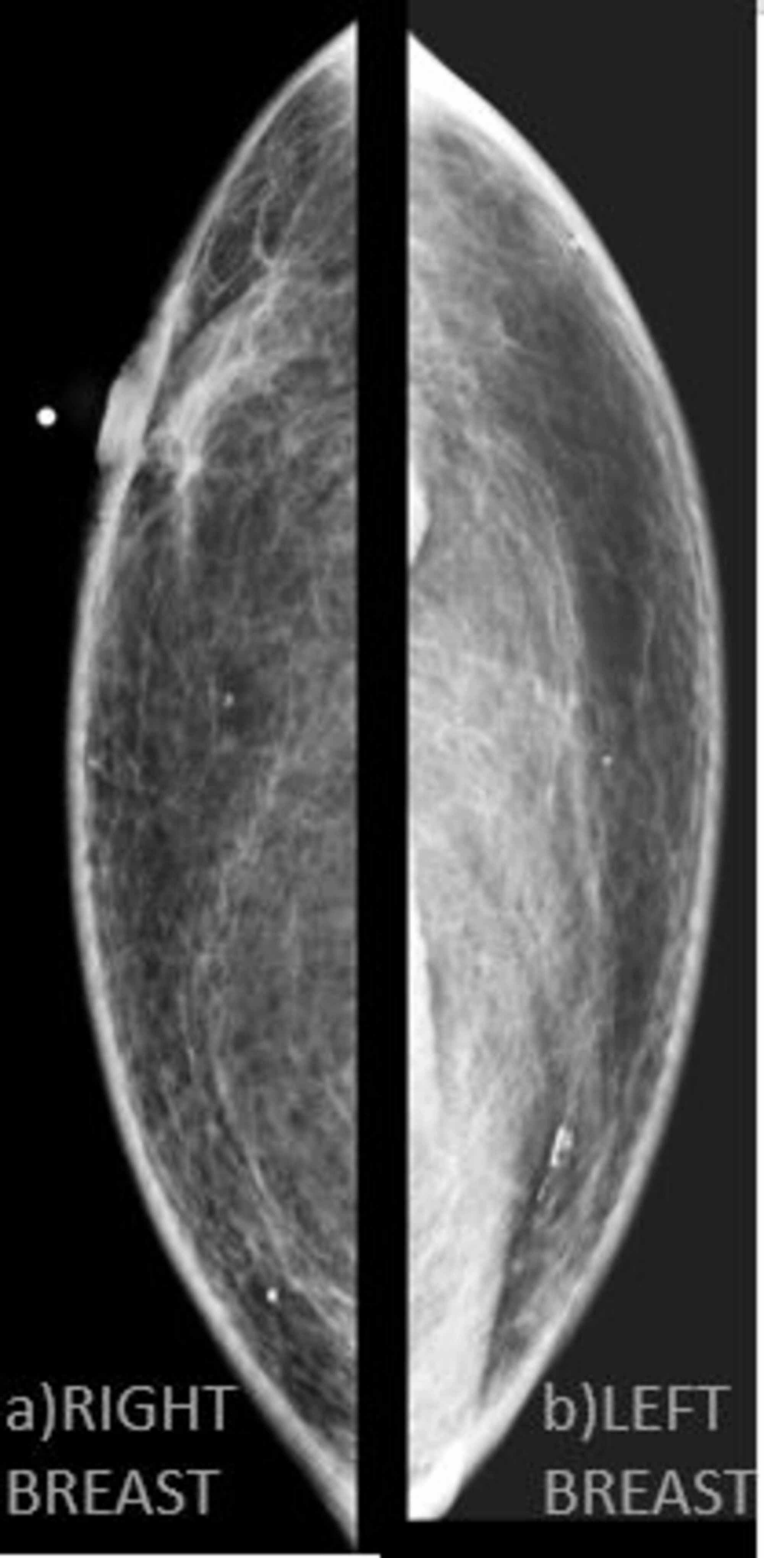
Mammography of the patient Mammography of the patient's breasts was not able to locate the mass; therefore ultrasound was needed to further understand our patient's condition. Right breast and left breast are denoted by grey text

The patient reported a history of a mass in this location for 10 years and a change in its size in the past two months. A primary breast malignancy was suspected, and follow-up breast consultation was recommended. At the initial presentation to the breast clinic, physical examination revealed a firm, tender, left subareolar mass with an irregular contour. Palpable axillary adenopathy was present on the ipsilateral side. Bilateral breast ultrasound and mammography were subsequently performed. Mammography of the right (Figure 2A) and left (Figure 2B) breast did not show the mass, thus indicating the need for further imaging.

**Figure 3:**
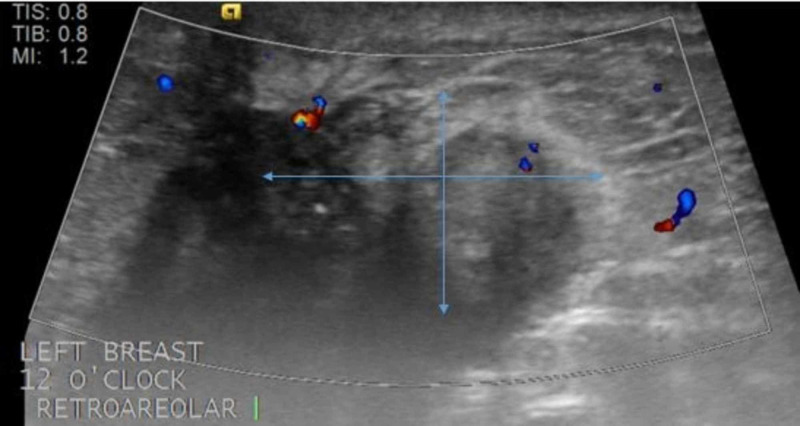
Ultrasound of the left breast in the transverse plane Targeted ultrasound demonstrates irregular mass with microlobulated margins measuring 27 x 17 x 21 mm at the site of the clinically palpable mass in the subareolar 12 o'clock region. Blue arrows outline the area of the mass

**Figure 4:**
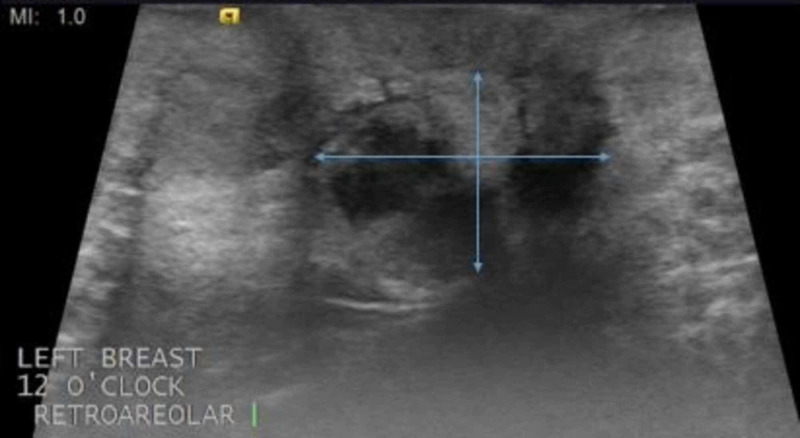
Ultrasound of the left breast in the longitudinal plane Ultrasound performed in the longitudinal plane again shows the mass; a biopsy is recommended for further evaluation. Borders of the mass are shown by the blue arrows

However, an ultrasound performed in the transverse plane (Figure 3) and in the longitudinal plane (Figure 4) showed the mass more clearly. 

**Figure 5:**
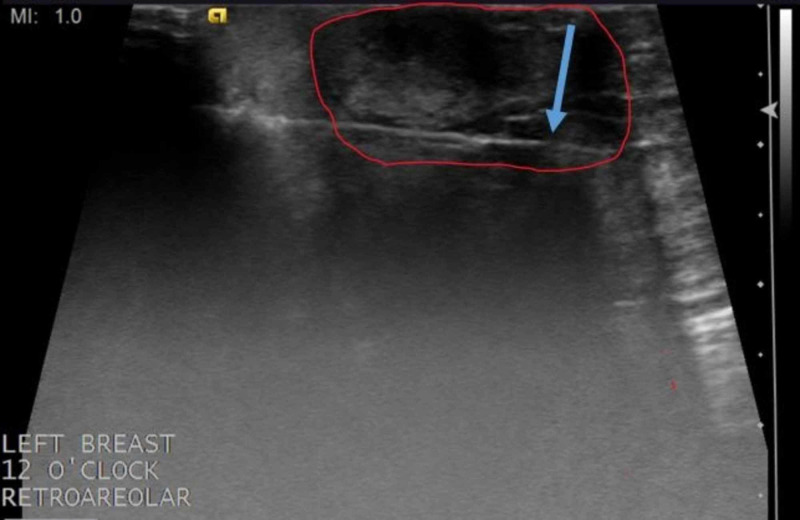
Biopsy of the left breast mass A biopsy was performed in the left breast and sent for evaluation. The biopsy needle indicated by the blue arrow was successfully able to obtain a sample from the mass circled in red

Exam of the left breast revealed a subareolar mass located at the 12 o’clock position measuring 27 mm and a left axillary lymph node measuring 13 mm. Based on these findings, a Breast Imaging Reporting and Data System (BI-RADS) score of 5 was deemed appropriate at this time. Ultrasound-guided needle core biopsies were obtained from the left breast mass (Figure 5) and the left axillary lymph node.

**Figure 6:**
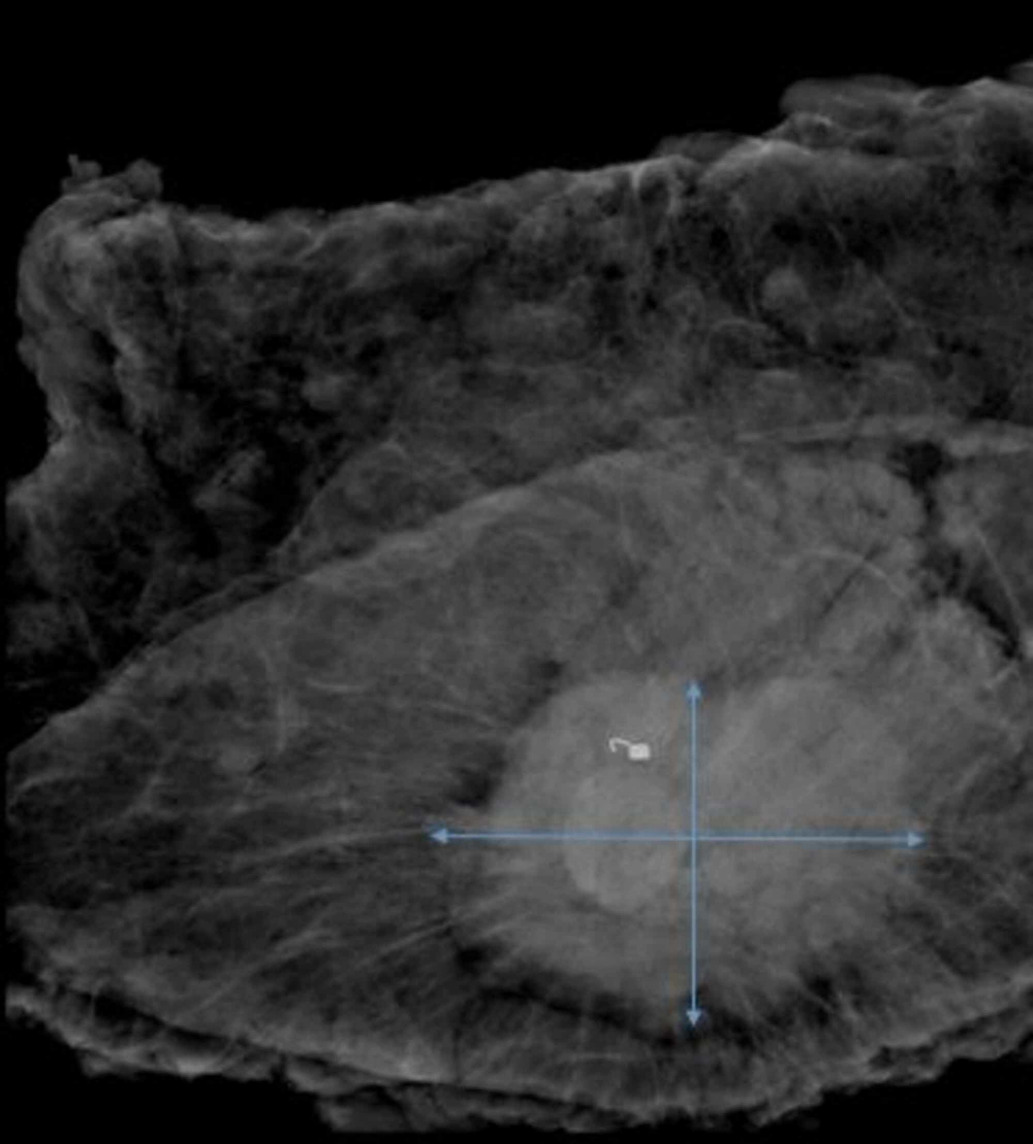
Left breast mastectomy surgical specimen Mass as seen after surgical removal. Blue arrows highlight the area of the mass

The breast mass was found to have histological findings consistent with invasive ductal carcinoma, and the axillary lymph node biopsy revealed metastatic ductal carcinoma. Cells obtained during biopsy revealed 99% of cells that were estrogen-receptor-positive, and 90% of cells that were progesterone-receptor-positive and HER-2-negative; 15% of cells were Ki-67-positive and E-cadherin-positive. Treatment options were discussed with the patient and a decision was made to perform a surgical resection. The patient was scheduled for a modified radical mastectomy of the left breast with left sentinel lymph node removal. The mass was then removed (Figure 6).

## Discussion

The most common form of breast cancer in men is invasive ductal carcinoma [3]. This specific pathology is characterized by initial development in the ducts of the breast followed by an invasion of surrounding tissues [4-5]. While male and female breast cancers have similar outcomes in terms of survival, differences exist in the manifestation of breast cancer seen in men and women of varying age groups. For example, hormone receptor expression is more likely to be positive in older male populations diagnosed with breast cancer [6]. There are also differences in physiologic aromatase activity and in the efficacy of aromatase inhibitors as a potential treatment option, as this specific treatment produces greater benefits in female patients [7]. Furthermore, males with breast cancer are found to have BRCA2 mutations more frequently than BRCA1 mutations [7]. In addition, breast malignancies diagnosed in men have been noted to have different metabolic cell processes in neoplastic cells from that of the female counterparts [7]. Thus, age and sex are significant factors to consider when determining a treatment plan for a patient.

Current treatment options available for breast cancer include surgery, radiation therapy, chemotherapy, and endocrine therapy, some of which can be coupled together as adjuvant treatments; selection from these options primarily depends on tumor histology and staging [8]. Chemotherapy in elderly patients requires additional considerations, including factors such as functional status, as these agents have more toxic effects on this population [9]. Adjuvant use of hormone therapy, such as tamoxifen, appears to improve survival outcomes in males with breast cancer [10]. However, it is not without risks. One possible side effect of hormone therapy is thrombus formation; therefore, patients should be assessed for thrombotic risk prior to the initiation of therapy [11]. Consequently, comorbidities must be carefully reviewed when choosing treatment options.

Guidelines published by the National Comprehensive Cancer Network (NCCN) outline appropriate treatment for women with hormone receptor-positive and HER-negative breast cancer depending on factors such as tumor histology, node positivity, and hormone receptor expression [12]. The application of these guidelines to our patient given his HER-2-negative status, singular lymph node involvement, and poor candidacy for chemotherapy would recommend the use of endocrine therapy following primary surgical resection. The guidelines do acknowledge, however, the lack of significant data to support the use of chemotherapy in patients over 70 years of age. Moreover, these guidelines were devised with regard to female patients. Thus, a decision to use chemotherapy should be based on clinician judgment. However, as noted above, older males are more likely to have positive hormone receptor expression similar to our patient and as mentioned prior, males, in general, seem to respond favorably to adjuvant hormone therapy, making tamoxifen an appropriate adjuvant treatment in this situation. The use of these measures and appropriate determination of receptor expression as recommended by the NCCN guidelines support the recommended treatment for our patient.

## Conclusions

There is a scarcity of data on which to base the treatment of male breast cancer in the elderly due to its rarity. In the future, more studies may be conducted that may contribute to the optimal care of elderly male breast cancer patients; however, unfortunately, currently there is insufficient data on which to base treatment. Therefore, aspects guiding treatment options, such as the increased likelihood of hormone receptor expression in elderly males, should be highly considered. This must also be balanced with the potential risks associated with each form of treatment. Naturally, the patient’s wishes must also guide the planning of care.
